# Communications to the Editor

**Published:** 1887-01

**Authors:** Herman Mynter

**Affiliations:** Copenhagen


### Communications to the Editor.

COPENHAGEN, Oct. 14, 1886.

*My Dear Doctor:*

Among the numerous hospitals in Copenhagen there is one which strikes the visitor as unique, partly on account of its purpose, partly on account of the munificence and elegancy with which it has been built and equipped by the city of Copenhagen. It is the epidemic hospital, also called the Bleydam's Hospital, from its location on the common pasture, the Bleydam. It is new, built during the years 1876 and 1883, and cost, completed and furnished, about 1,300,000 crowns, equal to about $350,000. The hospital ground contains about twenty acres, beautifully laid out as a garden. The hospital consists of an administration building, a kitchen, a laundry, a stable, a chapel, with rooms for post-mortem examinations, and eight cottages. Two of these cottages contain rooms with but one bed (twelve rooms in each), one used for patients who enter the hospital for observation, being under suspicion of epidemic diseases, the other for pay patients of a better class. The six cottages left are each divided into two parts with separate entrances, one for men, the other for women, and contains two wards of twelve beds each, and two single rooms of one bed each, besides a room for the nurse, an office, bathrooms, water closets, etc. The hospital proper, therefore, contains 180 beds in the different cottages, but besides these five felt-tents

(Dœcker's patent) are provided, containing thirty-seven beds, so that the hospital contains in all 217 beds, which number may be increased to 300 in case of necessity (cholera for instance.) There is in the hospital eighty-five square feet of floor and 1,038 cubic feet of air for each bed. The cottages are all removed at least eighty feet from the fence, and arranged in two rows of four cottages, on both sides of the kitchen and the laundry. The space between the cottages in the same row is fifty feet; the chapel is 350 feet from the nearest cottage.

The cottages are all one-story buildings, built of brick, with a slate Reiterdach roof. The six cottages are heated and ventilated by air of furnaces, very much like a Barstow furnace. The cold, fresh air is heated in a room surrounding the furnace, and enters from there the wards. The vitiated air is removed during the winter by aid of suction force through a chimney, the heat from the furnace furnishing the force, and during the summer by aid of the Reiterdach roof, the windows of which are opened on the side opposite the direction of the wind. On this side there is diminished atmospheric pressure and a current of air will, therefore, take place outwards.

The water closets and urinals are all trapped and of improved pattern (Marino's). In the two cottages with single rooms, each room is heated and ventilated by aid of a Krarup, ventilating stove. The purpose of the hospital is partly to treat patients with epidemic diseases, partly to act as a preventive to epidemics, by receiving suspicious cases for observation. It is built principally to receive cases of small-pox, exanthematic typhus, dysentery and cholera, but, as generally only sporadic cases of these diseases occur in Copenhagen, it receives, under common circumstances, all other diseases of epidemic nature, especially scarlatina, diphtheria, measles and erysipelas, cases of which are now denied entrance in all the other large hospitals, except in case of necessity, (tracheotomy, for instance).

From November, 1879, at which time the hospital was opened, to January, 1884, 1,926 patients were received, of which 935 were cases of scarlatina, with a mortality of twelve, six per cent.; 459 diptheria, with a mortality of six, one per cent.; 118 measles, with a mortality of seven, two per cent.; 94 variola, with a mortality of six, four per cent. No cases of cholera have been received during that time, and but few cases of epidemic dysentery and exanthematic typhus. A large number of tracheotomies have been performed with about twenty-five per cent. recoveries.

During the year 1884, 808 patients were treated in the hospital, of which 111 died; 103 patients had scarlatina, of which four died; 120 patients had diphtheria, of which five died; 178 patients had measles, of which fifty-five (all children) died, being a mortality of thirty-one per cent.; 56 children were treated for croup; in twelve of these the croup occurred as a complication to measles, and ten of these died, being 83 per cent.; of the 44 left, 30 died, being a mortality of 68 per cent.; 163 cases of erysipelas were treated, of which eleven died, being 6.7 per cent. mortality. Fifty-three per cent. of the patients received paid the hospital for all expenses incurred; the rest were treated and cared for at the city's expense.

The medical staff consists of a physician-in-chief (Dr. Med. S. Soerensen), one first assistant physician and three internes who change every two months with the internes at the commune hospital.

To prevent the spread of epidemic diseases from the hospital to the city, or from one ward to another, the following measures are employed.

The physicians, attendants and all visitors are required, before entering a cottage, to put on a linen duster and cap, which is kept and retained in the office of the cottage. Before leaving the cottage they are required to take off the duster and then to wash their heads and hands thoroughly. No attendant is allowed to leave the hospital for any purpose whatever without first taking a bath and changing the dress.

No visitors are allowed in the hospital, the only exception being the relatives of dying patients. They have then to use the same precautions as the attendants. The patients are not allowed to leave the hospital before they are incapable of spreading the contagion. During the four years mentioned eight patients contracted scarlatina in the hospital and six attendants were taken sick, three with variola, two with scarlatina and one with diphtheria.

To conclude, I will mention the city ordinances, which have a direct bearing upon the prevention of epidemic diseases. The board of health consists of five members—the superintendent of police, the mayor, the health physician and two members of the common council—elected for five years.

The superintendent of police is chairman and it is his duty to see that all laws and regulations are executed.

The city is divided into a number of districts, each with a city-physician, whose duties are partly to care for the sick poor, partly to watch over everything relating to the hygiene. Every physician, practicing in the city, is required by law to send a weekly statement to the health physician of all cases of contagious and epidemic nature in his practice. He is moreover required to give immediate notice of all cases of cholera, yellow fever, epidemic dysentery, exanthematic typhus, variola and pest, which diseases are always to be treated at public expense, and of scarlatina, diphtheria and typhoid fever, in the case that they are declared to be epidemic, and therefore to be treated at public expense.

It is the duty of every citizen, especially every landlord, house-owner, manufacturer, etc., to notify the police of every suspicious case known to him. The police then notifies the board of health, who then examines the case.

The board of health has power to order anybody, who suffers from the diseases mentioned, and who cannot be so isolated in his own home, that there is no danger of spreading the disease, to be sent to the epidemic hospital and stay there, till he cannot any longer communicate the disease. It has also the power to send dubious cases to the epidemic hospital for observation.

If the patient cannot be moved, the board of health may make such arrangements and take such precautions in regard to his care and isolation as it deems necessary.

It may order the tenants of houses, in which diseases are prevalent, to move out immediately, by paying the expenses. It may thereafter order the houses disinfected under its own observation or disinfect them itself, and keep them vacant as long as it deems necessary. It may order public and private schools closed; also theatres, etc. All expenses incurred by isolation, disinfection of houses, moving out of tenants, etc., are paid by the city; also all expenses incurred by the care and treatment of the diseased persons, if poor, or if sent to the epidemic hospital by order of the board of health.

I remain very truly yours,

HERMAN MYNTER, M. D.

